# The Skin-Mucus Microbial Community of Farmed Atlantic Salmon (*Salmo salar*)

**DOI:** 10.3389/fmicb.2017.02043

**Published:** 2017-10-20

**Authors:** Giusi Minniti, Live Heldal Hagen, Davide Porcellato, Sven Martin Jørgensen, Phillip B. Pope, Gustav Vaaje-Kolstad

**Affiliations:** ^1^Faculty of Chemistry, Biotechnology and Food Science, Norwegian University of Life Sciences (NMBU), Ås, Norway; ^2^Nofima AS, Norwegian Institute of Food, Fisheries and Aquaculture Research, Ås, Norway

**Keywords:** skin, mucus, teleost, microbiome, stress, aquaculture, *Salmo salar*

## Abstract

The skin of the teleost is a flexible and scaled structure that protects the fish toward the external environment. The outermost surface of the skin is coated with mucus, which is believed to be colonized by a diverse bacterial community (commensal and/or opportunistic). Little is known about such communities and their role in fish welfare. In aquaculture, fish seem to be more susceptible to pathogens compared to wild fish. Indeed common fish farming practices may play important roles in promoting their vulnerability, possibly by causing changes to their microbiomes. In the present study, 16S rRNA gene amplicon sequencing was employed to analyze the composition of the farmed *Salmo salar* skin-mucus microbiome before and after netting and transfer. The composition of the bacterial community present in the rearing water was also investigated in order to evaluate its correlation with the community present on the fish skin. Our results reveal variability of the skin-mucus microbiome among the biological replicates before fish handling. On the contrary, after fish handling, the skin-mucus community exhibited structural similarity among the biological replicates and significant changes were observed in the bacterial composition compared to the fish analyzed prior to netting and transfer. Limited correlation was revealed between the skin-mucus microbiome and the bacterial community present in the rearing water. Finally, analysis of skin-mucus bacterial biomasses indicated low abundance for some samples, highlighting the need of caution when interpreting community data due to the possible contamination of water-residing bacteria.

## Introduction

The body surface of vertebrate animals represents a physical barrier between the environment and the animal host. Skin protects the host from the entry of pathogenic organisms or allergens, but also from the leakage of water, solutes or nutrient (Ángeles Esteban, 2012). The skin of teleost is different from that of mammals because it secretes mucus, which exhibits immune functions (Salinas et al., 2011). Mucus contains mucins (heavily *O*-glycosylated proteins) (Barchi, 2013), and an array of antimicrobial compounds like immunoglobulins, antimicrobial peptides and different enzymes (Ángeles Esteban, 2012). The presence of a mucosal tissue on fish skin represents an evolutionary adaption to the water environment (Xu et al., 2013), which is populated by a large number of potentially harmful organisms (Magnadottir, 2010). Indeed, mucus represents the first barrier against infectious pathogens (Tort et al., 2003). All the mucosal surfaces of humans and animals are colonized by different bacterial species (commensal and/or opportunistic), which play a key role in the development of the host immune system (Cebra, 1999; Lee and Mazmanian, 2010; Maynard et al., 2012). In contrast to the well-studied human skin microbiome (Schommer and Gallo, 2013; Dorrestein et al., 2016; Gallo, 2017) only a limited number of studies have focused on the complexity of the bacterial community associated with the fish skin-mucus (Llewellyn et al., 2014; Chiarello et al., 2015; Lowrey et al., 2015; Lokesh and Kiron, 2016; Kearns et al., 2017). Interestingly, even though the majority of bacteria detected in the skin-mucosal surfaces belong to the phylum *Proteobacteria*, high variations at the species levels have been observed in the abovementioned studies. For instance, Chiarello et al. (2015) reported variability of the skin-associated community between host species, individuals and as well among different external body parts. Moreover, when comparing the microbial community of skin, gills, olfactory rosettes and anterior and posterior gut tissues from rainbow trout, Lowrey et al. (2015) observed that the highest microbial diversity was found in the external mucosal sites of fish.

The ability of fish to maintain a healthy balance between commensal and opportunistic bacteria in their skin-mucus is suggested to represent a key factor to preserve fish health (Gómez and Balcázar, 2007). Unfortunately, the healthy balance of the microbiome can be altered by disturbance factors such as stress or antibiotics (Boutin et al., 2013; Carlson et al., 2015). In aquaculture of salmonids, stressful events include (and are not limited to) netting, sorting and transport (Iversen and Eliassen, 2009). These practices could potentially affect the balance of the fish-skin microbiome and reduce the bacterial biodiversity, promoting proliferation of opportunistic bacteria, a process well documented in mammals (Myers, 2004; Jones et al., 2014; Tomasello et al., 2016). Moreover, a previous study (Iguchi et al., 2003) evaluated the effect of stress on the immune system of fish and reveled an increase of disease susceptibility due to immunosuppression, confirming the close relation existing among stress, immune system responses and pathogens. Stress in aquaculture is considered maladaptive when disturbances cause a prolonged stress response, which is harmful to the fish welfare (Barton, 2002; Llewellyn et al., 2014). Recent studies on the bacterial taxa living in the skin-mucosal surfaces of fish reported a shift in the microbiome as a consequence of exposure to prolonged stress, enhancing the growth of potentially pathogenic bacteria (Boutin et al., 2013; Sylvain et al., 2016). Farmed aquatic animals are often exposed to maladaptive conditions and diseases. Skin disorders represent one of the problems associated with fish mortality in aquaculture. It is estimated that 1.1–2.5% of farmed fish die due to ulceration (Karlsen et al., 2017). Therefore, understanding the composition of the skin-mucus microbiome of farmed fish may represents a step toward improving the welfare of species such as *Salmo salar*. Despite the importance of this topic, little is known regarding the host-associated bacterial population present in the skin-mucus.

In the current work, 16S rRNA sequencing analysis has been used to study the microbial community present in the skin-mucus of farmed *Salmo salar* and the potential influence of common aquaculture practices, such as fish netting and transfer, on its composition. In addition, the bacterial community present in the rearing water was also monitored during all experiments to compare its similarity with the salmon skin-mucus microbiome.

## Materials and Methods

### Fish and Sampling Procedure

**FIGURE 1:**
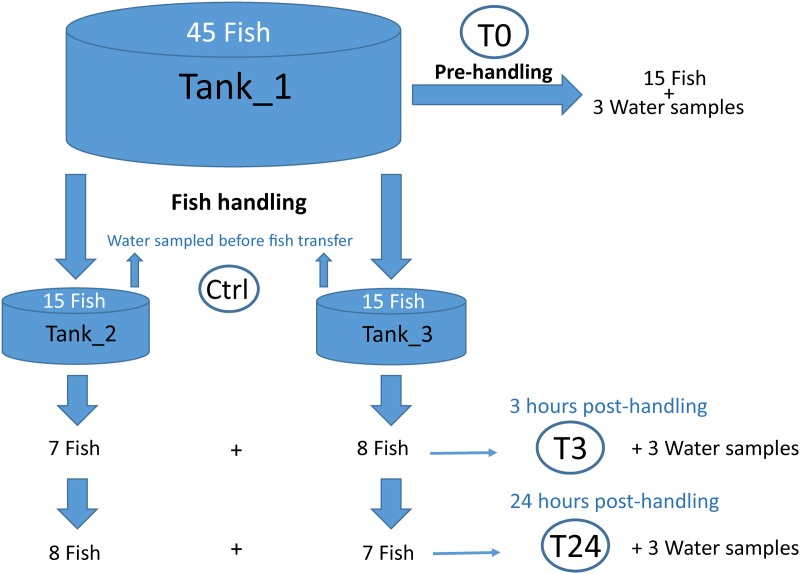
Schematic illustration of the experimental design. A total of 45 fish were used in this experiment. Fifteen fish were sampled directly from the main tank (Tank_1) and skin mucus samples were collected (T0). The resting 30 fish, after netting, were transferred into Tank_2 and Tank_3, and successively mucus samples were collected at 3 h (T3) and 24 h (T24) post-handling. In parallel, water samples were collected at different time points, with 3 replicates taken per tank for each time point. Rearing water from Tank_2 and Tank_3 was additionally sampled before fish were transferred into the new tanks (Ctrl).

Forty five seawater-adapted post-smolt *Salmo salar* (±300 g each) from the Nofima research center NCRA in Sunndalsøra, Norway were randomly selected for this study. A schematic overview of the experimental sampling plan is illustrated in **Figure 1**. At the time of sampling, salmon had been kept in Tank_1 for 6 months, and the total biomass of the tank was approximately 96 kg/3.3m^3^. Fish were fed with Ewos Opal 200, following a feeding regime of 6 times/hour, with 8 s feeding/time. The source of the water utilized in the experiment was seawater from a depth of 40 m mixed with fresh ground water, following filtration and UV disinfection (32 ppt salinity and temperature around 10°C). The tank based-system was a Recirculation Water System (RAS). Fifteen of the forty five fish were sampled directly from Tank_1, representing the pre-handling time point (T0), killed with an overdose of MS-222 and immediately transferred to the lab. Mucus samples were taken from the right side of the fish, over the entire side, using sterilized swabs (Plain swab sterile wooden applicator cotton tipped, Copan, Italy) and stored at -80°C until further analysis. The remaining 30 fish were transferred to a small tank containing the same water as Tank_1, lifted up simultaneously with a sterilized net, kept in air for 30 s and back in water to recover; the process was repeated three times. After netting, fish (15 fish per tank) were transferred into Tank_2 and Tank_3, which served as technical replicates. All the tanks used in the experiment had a flow through system. The inlet water to each single tank was the same but the water was not shared among them. The fish feeding was interrupted after fish handling to avoid microbial contamination from unconsumed food as it is observed that fish tend to fast after stressful events. Fish were sampled from Tank_2 and Tank_3 after 3 h (T3) and 24 h (T24) post-handling (15 fish each time), using the same sampling and mucus processing procedure described previously. Furthermore, 50 ml of water was collected from all the tanks, at all experimental time points, using sterile 0.2 μm hollow fiber syringe filters (Dyna Gard, Microgon Inc., Laguna Hills, CA, United States) to retain the bacteria present in the water (3 replicates per tank). Filters were stored at -80°C until further analysis. Samples were entitled according to the source of the sample (water; “W” or mucus; “M”), time of collection (pre-handling ; “T0,” 3 h post-handling; “T3” or 24 h post-handling; “T24”) and sample tank (Tank 1–3), e.g., sample M2-T3-3 represents mucus sample number 2 collected from Tank_3 at 3 h post-handling. The animal experiment was approved and done according to laws and regulations of the Norwegian Food Safety Authority and the ‘European Convention for the Protection of Vertebrate Animals used for Experimental and other Scientific Purposes’ (EST 123).

### Samples Preparation and 16s rRNA Gene Sequencing (rrs)

DNA was extracted from mucus and water filters with the DNeasy tissue kit (Qiagen, Germany), following the protocol for Gram positive bacteria with some modifications. Achromopeptidase was utilized (incubation for 1 h at 37°C) in the first step of the extraction process, ensuring the lysis of Gram positive bacteria (Ezaki and Suzuki, 1982). Proteinase K (40 μl) and ATL buffer (180 μl) were added to the samples, and tubes were incubated at 55°C for 1 h. Successively, 200 μl of AL buffer was used as last lysis step (incubation at 70°C for 10 min). The manufacture’s protocol was followed during the remaining steps. The DNA extracted was stored at -20°C. Sample preparation for 16S rRNA sequencing analysis by Miseq was performed according the Illumina guide (16S Metagenomic Sequencing Library Preparation, Part. 15044223 Rev A). A primer set targeting the V3-V4 hypervariable regions, Pro341F (5′-CCTA CGGGNBGCASCAG-3′) and Pro805R (5′-GACTACNVGGGT ATCTAATCC-3′) (Takahashi et al., 2014) were used to amplify 16S rRNA genes. PCR amplification was performed using the polymerase iProof High-Fidelity (Bio-Rad, Hercules, CA, United States) with the following cycle conditions: initial denaturation at 95°C for 3 min, followed by 35 cycles of 95°C for 30 s, 55°C for 30 s, 72°C for 30 s, and concluded by a final extension at 72°C for 5 min. The quality of the amplicon DNA was checked by agarose gel electrophoresis. Sample concentrations after amplicon PCR and cleaning steps were quantified using Qubit dsDNA HS Assay (Invitrogen). Miseq (Illumina) was used to sequence mucus and water samples. PhiX Control library was combined with the amplicon library (expected at 15%).

### Bioinformatics and Statistics

The pipeline Usearch v.8.1861 (Edgar, 2010) implemented in QIIME v1.8.0 (Kuczynski et al., 2012) was used to analyze the data (all executed command lines are listed in Supplementary Materials and Methods). First, paired reads were merged, quality filtered (E_max = 1) and trimmed (430 nucleotides) to ensure the presence of sequences of sufficient quality. Chimeric sequences were identified and excluded from the dataset using the command cluster_otus in Usearch. The same command (cluster_otus) performs 97% Operational Taxonomic Units (OTU) clustering using the UPARSE-OTU algorithm, thus it was used to construct the set of representative OTUs. Finally, taxonomy was assigned using the UTAX algorithm (Edgar, 2010) with the full-length RDP training set (utax_rdp_16s_trainset15), enabling the construction of the OTU table. Filter_otus_from _otu_table.py in QIIME was used to filter out OTUs making up less than 0.005% of the total, by using default parameters and –min_count_fraction set to 0.00005 (Bokulich et al., 2013). The samples were further normalized to the smallest library to remove sample heterogeneity. The generated OTU table was utilized to create the taxonomy plots and to construct the phylogenetic tree, which was subsequently used to generate the unweighted and weighted UniFrac distances by QIIME. The OTU table was also used to calculate the Bray-Curtis distance matrix using the R (version 3.2.3), package Vegan (Oksanen et al., 2013). Principal Coordinate Analysis (PCoA) of unweighted and weighted UniFrac matrix (Lozupone and Knight, 2008) were performed to assess phylogenetic distances between water and mucus microbiome at different sampling times (T0, T3, T24, Ctrl). PCoA analysis was also performed on the Bray-Curtis distance matrix (Beals, 1984). The α-diversity of mucus and water samples at T0, T3, T24 and Ctrl were calculated at OTU level using Shannon index. The Dunnett-Tukey-Kramer Pairwise Multiple Comparison Test Adjusted for Unequal Variances and Unequal Sample Sizes (Lau, 2013) was used to compare the bacterial diversity, measured by Shannon index, among groups of samples. Finally, Permutational Multivariate Analysis of Variance (Adonis) (Anderson, 2001) using the weighted UniFrac distance matrix in R, package Vegan, was utilized for significance testing on the water and mucus samples.

### Droplet Digital PCR Reaction and Data Analysis

The QX200 Droplet Digital PCR (ddPCR^TM^) System (Bio-Rad, Munich, Germany) was used to quantify the number of copies per microliter of the extracted DNA from mucus and water samples. The ddPCR reaction contained 10 μl of Eva Green Super Mix (Bio-Rad, United States), 100 nM of each primer (Pro341F-Pro805R, see above) and 2 μl of template DNA. In addition, sterile nuclease-free water (VWR) was included in the ddPCR reaction to reach a final volume of 22 μl. A volume of 20 μl of the ddPCR reaction was used to generate 40 μl of droplets using the QX100 droplet generator (Bio-Rad, Munich, Germany). Droplets were transferred to a 96-well plate and amplified with the following conditions: initial denaturation at 95°C for 5 min, followed by 45 cycles at 95°C for 30 s, 53°C for 30 s, 72°C for 45 s and stabilization signal at 4°C for 5 min and 90°C for 5 min. Afterward, droplets were analyzed in the QX200 droplet reader (Bio-Rad, Munich, Germany). Mucus and water samples were studied in triplicates and all the data were analyzed by the QuantaSoft software 1.7 (Bio-rad, Munich, Germany), which provides the concentration values of the ddPCR target DNA in copies/μl. Successively, the numbers of bacterial copies/μl present in the starting samples were calculated: The concentration given by the QuantaSoft software multiplied by the reaction volume and divided by the volume of the starting material added in the PCR (Bio-Rad Droplet Digital PCR Applications Guide, Bullettin 6407, Rev A).

### Nucleotide Sequence Accession Numbers

Sequence data are available at NCBI Sequence Read Archive under accession number SRP107063.

## Results

**FIGURE 2:**
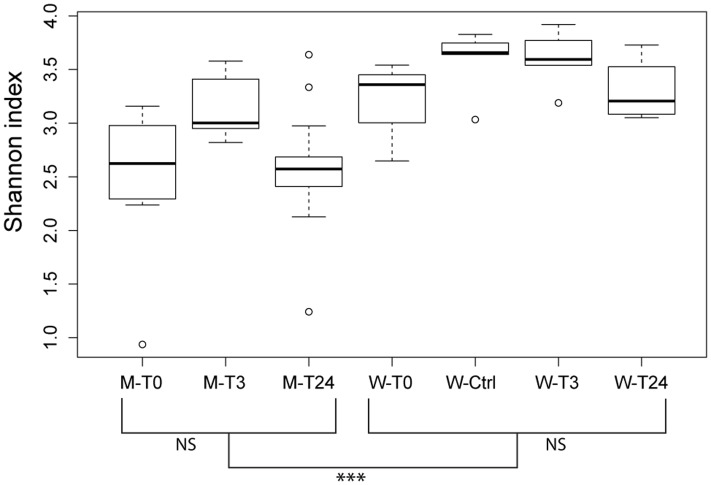
Community diversity estimations by Shannon index at an OTU level. Each bar describe mucus or water samples collected at the different time points (T0, T3, T24 + Ctrl water). Significant differences among the water samples are calculated using the Dunnett-Tukey-Kramer Pairwise Multiple Comparison Test Adjusted for Unequal Variances and Unequal Sample Sizes. Significance degree is represented with stars; *P* < 0.05 with one star (^∗^); *P* < 0.01 with two stars (^∗∗^); *P* < 0.001 with three stars (^∗∗∗^). No significance is indicated as NS.

**FIGURE 3:**
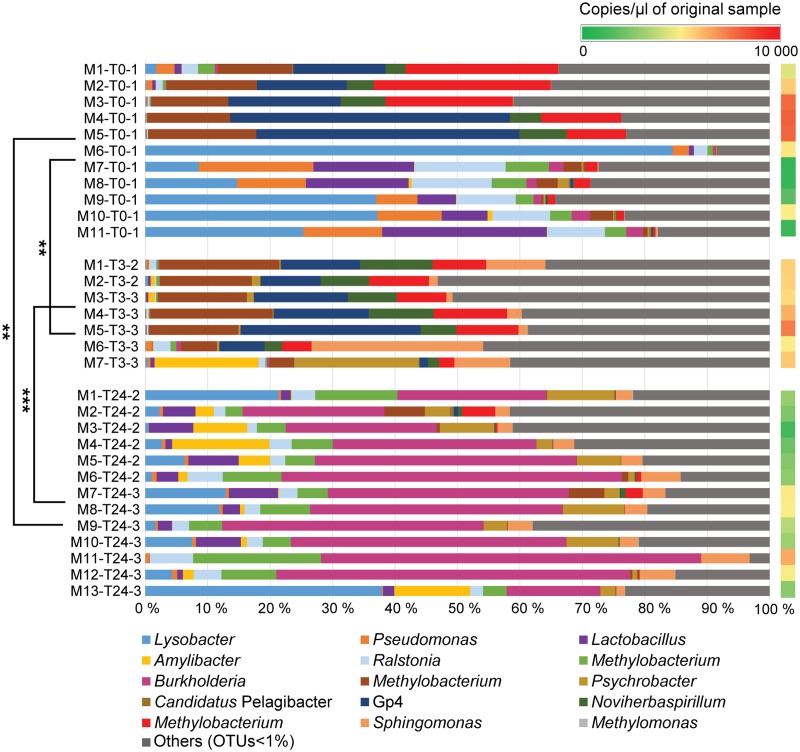
Relative abundances of bacterial genera identified in mucus samples. The bar chart shows relatively abundant genera inherent to the skin mucosal microbiome at the different time points (T0, T3, T24). The OTUs with relative abundance values greater than 1% of the total sequences are mainly considered in the barchart. The OTUs <1% of the total sequences are assembled together as “Others (OTUs<1%)”. A comprehensive summary of the taxonomic groups is given in Supplementary Table S4. Permutation Multivariate Analysis of Variance using weighted UniFrac distance matrix calculated from the total OTU dataset are performed among the different experimental time points (shown on the left) (see Supplementary Table S3). Significance degree is represented with stars; *P* < 0.05 with one star (^∗^); *P* < 0.01 with two stars (^∗∗^); *P* < 0.001 with three stars (^∗∗∗^). The color gradient (shown on the right) illustrates the DNA concentration of mucus samples (copies/μl) detected by Droplet Digital PCR (see Supplementary Table S2).

**FIGURE 4:**
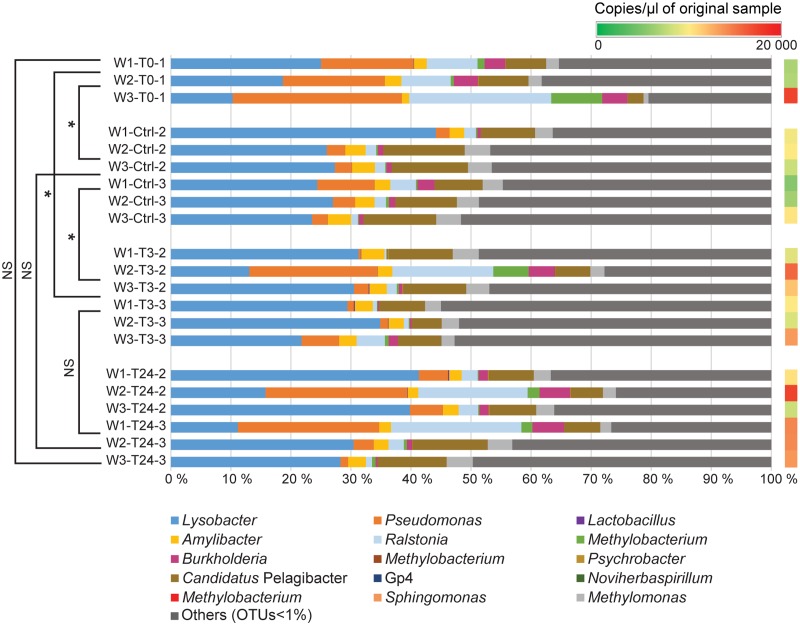
Relative abundance of bacterial genera identified in the rearing water. The bar chart shows the tank water microbiome (3 replicates per tank) at the different time points (T0, T3, T24 + Ctrl water). The OTUs with relative abundance values greater than 1% of the total sequences are mainly considered in the barchart. The OTUs <1% of the total sequences are assembled together as “Others (OTUs<1%). A comprehensive summary of the taxonomic groups is given in Supplementary Table S4. Permutation Multivariate Analysis of Variance using weighted UniFrac distance matrix calculated from the total OTU dataset are performed among the different experimental time points (shown on the left) (see Supplementary Table S3). Significance degree is represented with stars; *P* < 0.05 with one star (^∗^); *P* < 0.01 with two stars (^∗∗^); *P* < 0.001 with three stars (^∗∗∗^). No significance is indicated as NS. The color gradient (shown on the right) illustrates the DNA concentration of water samples (copies/μl) detected by Droplet Digital PCR (see Supplementary Table S2).

16S rRNA gene sequencing was used to identify the bacterial composition and diversity of the skin-mucus microbiome of *Salmo salar* before and after fish handling. Samples from a total of 45 fish were originally collected. However, 15 of these samples did not generate PCR products, and were thus omitted from the analysis (Supplementary Table S1). The bacterial community in the tank water was additionally characterized to evaluate its influence on the skin-mucus microflora. After quality filtration, a total number of 2690828 reads were obtained from water (21 samples) and mucus (31 samples). The mean of read counts was 51746.692, with the highest values being 98187 (sample W3-T0-1) and the lowest being 22070 (sample W3-Ctrl-3) (Supplementary Table S1). Normalization of the dataset generated a total of 1147640 quality-filtered reads from the 52 samples analyzed. These sequences were assigned to 616 OTUs, which were utilized to construct the phylogenetic tree and generate unweighted and weighted UniFrac and Bray-Curtis distance matrices. Alpha diversity estimates represented by Shannon index indicated that the bacterial diversity was significantly higher in water samples compared to mucus (**Figure 2**). In order to focus on the most abundant taxa, **Figures 3, 4** visualize mainly the OTUs represented by greater than 1% of the total reads. The OTUs <1% of the total sequences are grouped together as “Others (OTUs<1%)”. An overview of the phylogenetic distribution of all the OTUs presented at class level is provided in Supplementary Figure S1.

### Species Variation Observed in Skin Microbiomes Prior Fish Handling

The microbial profile analysis of mucus from a total of eleven fish was performed at T0. Quantitative Droplet Digital PCR revealed variation in concentration of copies of 16S rRNA gene sequences among the individual fish (**Figure 3** and Supplementary Table S2). Three phyla were mainly observed on the salmon skin-mucus before handling, with *Proteobacteria*-affiliated phylotypes the most abundant followed by *Firmicutes* and *Acidobacteria*. The most abundant OTUs (≥1%) obtained at T0 were classified at genus level (**Figure 3**), illustrating intraspecies variation among the biological replicates. For instance, *Lysobacter*-affiliated phylotypes were the most abundantly observed in samples M6-T0-1 (84%), M9-T0-1 (37%) and M10-T0-1 (37.5%), while almost absent in samples M2-T0-1, M3-T0-1, M4-T0-1 and M5-T0-1. On the contrary, Gp4-affiliated phylotypes (uncultured bacteria from the phylum *Acidobacteria*) were mostly observed in samples M1-T0-1 (15%), M2-T0-1 (14%), M3-T0-1 (17%), M4-T0-1 (45%), and as well in M5-T0-1 (41.5%). In addition, *Pseudomonas*, *Noviherbaspirillum* and *Burkholderia* (the latter in low abundance) were detected in some of the samples, while OTUs affiliated with the genera *Ralstonia*, *Lactobacillus* and *Methylobacterium* were observed in almost all biological replicates with fluctuations in relative abundance.

### Skin Community Profile and Diversity Post-handling

**FIGURE 5:**
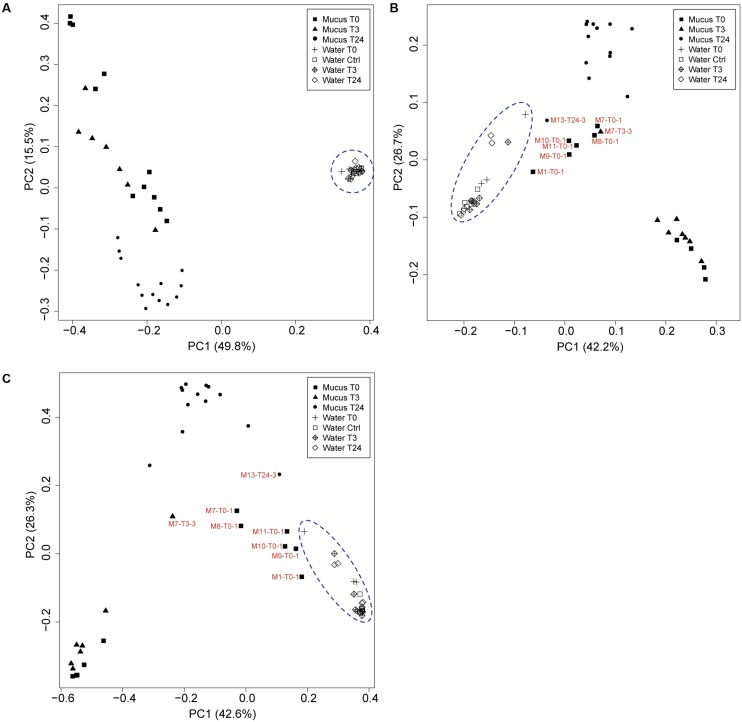
Principle coordinate analysis (PCoA) using the **(A)** unweighted and **(B)** weighted UniFrac distance matrices and **(C)** Bray-Curtis distance matrix between mucus and water associated microbiomes at the different time points (T0, T3, T24 + Ctrl water). Samples that are represented by low biomass (DNA concentration: Supplementary Table S1, ddPCR: Supplementary Table S2) and have a close correlation with water are indicated.

The same phyla detected in the skin microbiome at T0 were also observed in the samples collected from the handled fish in the two replicate tanks (Tank_2 and Tank_3 at T3 and T24). At T3, the genera-level population of the most abundant phylotypes seemed to be consistent across the assessed biological replicates and the community showed a similar profile as some of the fish sampled at T0 (**Figure 3**). However, intraspecies variation was still observed, with sample M7-T3-3, portraying a different proportion of abundant OTUs compared the other fish sampled at the same time point. Mucus collected at 3 h post-handling (T3) included seven samples, while samples taken at 24 h post-handling included thirteen mucus samples (Supplementary Table S1). Quantitative Droplet Digital PCR (ddPCR) highlighted variation in concentration of copies of 16S rRNA gene sequences within the individual fish at the same experimental time point and among samples collected at the different time points (**Figure 3** and Supplementary Table S2). The skin microbiome of fish collected at T24 showed a shift of the abundant OTUs and had lower intraspecies variation compared to the skin-mucus microbiome detected at T0 and T3 (**Figure 3**). For instance, the average of the relative abundances associated with the genera *Methylobacterium*, Gp4 and *Noviherbaspirillum* showed higher abundance before handling and at 3 h post-handling compared to 24 h post-handling. On the contrary, *Lysobacter* and *Lactobacillus*, which were abundant in some of the samples at T0, were again detected after 24 h post-handling. The most evident shift of the skin-mucus community was associated with the genus *Burkholderia*, which was present in small amount at T0 (1%) and T3 (below 1%), followed by a considerable increase after 24 h (38%). Significant differences existing among T0, T3, and T24 were supported by the beta-diversity distance matrices (UniFrac and Bray-Curtis), which validated the aforementioned intra-animal variation of the total bacterial community and illustrated that the fish mucus microbiomes before and after fish handling were phylogenetically distinct (**Figure 5**). Significant differences existing among T0, T3 and T24 were statistically corroborated by Permutational Multivariate Analysis of Variance (Adonis) (**Figure 3** and Supplementary Table S3).

### Water Community Profile and Its Comparison with the Skin-Mucus Microbiome

Water samples were collected from all tanks at the different fish sampling time points, and were subjected to the same *rrs* analysis pipeline to compare its similarity with the fish mucus microbiome. Additional water samples were collected from Tank_2 and Tank_3 before transferring the fish (Ctrl) to evaluate any differences with the original water containing the fish in Tank_1 (water T0) and the water to which the fish were transferred and assessed after 3 h and 24 h. Quantitative Droplet Digital PCR (ddPCR) indicated, in overall, a higher number of copies of 16S rRNA gene sequences in the water samples compared to the mucus samples and highlighted variation in concentration among samples, tanks and time points (**Figures 3, 4** and Supplementary Table S2). The most abundant OTUs (greater than 1%) in each sample were consistently observed in both technical replicates (Tank_2 and Tank_3) and all rearing water samples originating from the different time points (**Figure 4**). *Lysobacter* was the most abundant genus, followed by *Pseudomonas* and *Ralstonia*. Beta-diversity metrics (UniFrac and Bray-Curtis) (**Figure 5**), showed a clear variation between the water and mucus communities, which were visualized by two distinct clusters (outcome supported statistically by Permutational Multivariate Analysis of Variance, Supplementary Table S3). However, the mucus samples M6-T0-1, M7-T0-1, M8-T0-1, M9-T0-1, M10-T0-1, M11-T0-1, and M13-T24-1 exhibited a different trend and showed a closer correlation with the water samples (**Figure 5**).

## Discussion

In this study, we have used 16S rRNA gene sequencing to investigate the composition of the skin-mucus microbiome of farmed *Salmo salar* and the potential influence of common aquaculture practices on the microbial community. For fish that were sampled and analyzed before netting and transfer, the taxonomic analysis at phylum level exhibited a predominance of *Proteobacteria*, which was in agreement with previous studies conducted on different species of teleost (Wilson et al., 2008; Chiarello et al., 2015). Inspection of the data at genus level indicated differences in the bacterial community among the biological replicates (**Figure 3**). Individual variability has been reported for both wild and captive teleost and cetaceans (Larsen et al., 2013; Apprill et al., 2014). However, the variability observed at T0 cannot be conclusively addressed by this study, due to the limited number of biological replicates and the low cell biomass detected in several samples. The number of 16S rRNA gene copies obtained from the skin-mucus varied between individuals (**Figure 3** and Supplementary Table S2), showing low biomass in some samples, which may be related to low bacterial biomass in the mucus (Austin, 2006), technical challenges in the DNA extraction procedure or a combination of these. This may explain why amplification of the 16S rRNA genes was problematic in several samples (also observed by Lowrey et al., 2015). Notably, some of the mucus samples with the lowest biomasses (M7-T0-1, M8-T0-1, M9-T0-1, M11-T0-1) showed similarity with the microbial profile of the rearing water. This similarity is evident when comparing the genera distribution in **Figures 3 and 4** as well as the beta diversity analysis (**Figure 5**), where the abovementioned samples are clustering in close proximity with the water samples. It is therefore tempting to speculate that the low-biomass samples are partly represented by the microbiome present in rearing water, in addition to the mucus microbiome. Unfortunately, there is no appropriate way to sample skin-mucus without collecting some of the water associated with it. Our observations highlight the importance of quantifying the sample biomass using sensitive method like ddPCR to thereby determine the bacterial biomass and to ensure a correct interpretation of the microbiome profile, recognizing the potential bias from co-sampling of the surrounding environment.

The physiological response of the fish skin to stress has barely been scientifically studied, but it is generally accepted that mucus production increases upon stress events and the general immunologic state of the skin is altered (Tort et al., 2003). Similar responses have been documented in other mucosal systems such as the mouths of mammals, where expression of mucins are increased after stress (Bosch et al., 2000), highlighting the protective role of mucus. The common aquaculture practices such as netting and transfer may cause fish stress and removal of mucus, allowing the growth of potential opportunistic bacteria. For instance, it was observed that a damaged mucus layer caused high mortality in salmonids during challenge experiments with bacteria (Svendsen and Bøgwald, 1997; Madetoja et al., 2000). In the present study, the comparison of the host microbiome before and after handling showed a shift in the composition of the community after 24 h (**Figure 3**). This was supported over both technical replicates (Tank_2 and Tank_3) by the statistic test Adonis (Supplementary Table S3). The most prominent change observed in the microbiome over the 24 h post-handling period, was the rise of the genus *Burkholderia*. The order *Burkholderiales* has also been detected as component of the skin-mucus microbiome of rainbow trout (*Oncochynchus mykiss*) and cow-nose rays (*Rhinoptera bonasus*) (Lowrey et al., 2015; Kearns et al., 2017). In particular, the genus *Burkholderia* is known to either have a beneficial or pathogenic relationship with other host organisms (Compant et al., 2008), but is mostly documented as a pathogen for plants (Jeong et al., 2003), humans (Valvano, 2006) and animals (Whitlock et al., 2007). To the best of our knowledge, this genus has not been linked to pathogenesis in fish. In addition to the increased relative abundance of *Burkholderia*-affiliated phylotyopes at T24, a decrease of *Methylobacterium*-affiliated phylotypes was observed in almost all biological replicates. Since *Methylobacterium* has mostly been detected in healthy fish, the decline of this genus may be associated with an increase of opportunistic bacteria. It is well documented that *Methylobacterium* spp. are able to produce poly-β-hydroxybutyrates, which are recognized to inhibit the growth of pathogens in other host-bacterial communities (Defoirdt et al., 2007; Halet et al., 2007; Boutin et al., 2013). It is also well known from other studies that stressful conditions change the microbial profile of the mucosal surfaces, leading to microbial imbalance (Boutin et al., 2013; Sylvain et al., 2016). The shift observed at T24 in the present study may represent the beginning stage of dysbiosis, but in the absence of a negative control (fish not exposed to netting and transfer at T3 and T24) a firm conclusions cannot be drawn. The inclusion of a true negative control was not possible due to logistic issues in the experimental setup. The fish stocking level in Tank_1 (96 kg/3.3m^3^) differed substantially from the stocking level in Tank_2 and Tank_3 used for hosting the fish at T3 and T24 (4.5 kg/0,5m^3^). Therefore, given the large discrepancy in fish density among the experimental tanks, the use of fish from Tank_1 as negative control would not be possible. Nevertheless, our data demonstrate for the first time that the Atlantic salmon skin microbiome can change substantially within only 24 h.

Another important aspect of this work was to investigate the similarities and diversities between the bacterial communities found in the rearing water and the skin-mucus microbiome. Analysis at the genus level of the most abundant phylotypes (**Figure 4**) illustrated similarities between water samples collected in the different tanks and at the three experimental time points. Many genera detected in rearing water were also observed in mucus such as *Lysobacter* and *Ralstonia*, in accordance with the hypothesis that fish skin mucosal microbiome is colonized by strains living in the surrounding environment (Horsley, 1973). However, as mentioned above, the low biomass observed in some of the mucus samples may have distorted the results, causing detection of members of the water microbiome in the mucus. Despite the similar genera distribution observed between the two environments, beta-diversity analysis (UniFrac and Bray-Curtis; **Figure 5**) showed distinct phylogenetic distances between water and mucus derived microbiomes. These results were statistically confirmed by Adonis (Supplementary Table S3), suggesting that each microbial community is adapted to its own environment. A similar outcome has been observed in another study on Brook Charr (*Salvelinus fontinalis)*, where the role of stress on the skin-mucus microbiome was investigated using 16S rRNA gene sequencing analysis (Boutin et al., 2013). The study by Boutin et al., demonstrated that the water community shared common genera with the fish skin microbiome, although UniFrac analysis indicated significant differences between the two microbiomes. Thus, despite the skin-mucus microbiome is in direct contact with the external environment, it seems to be specifically adapted to the salmon mucus. For instance, a study conducted by McKenzie et al. (2012) showed that the skin bacteria community present on amphibians is species specific, even when different amphibian species co-exist in the same pond.

## Conclusion

Our data suggest that netting and transfer of fish between tanks represent a potential cause for the rapid rise of Burkholderia (and decline of others). However, the lack of additional controls (fish not exposed to netting and transfer) at T3 and T24 does not allow us to rule out other influences such as the fish’s acclimation to the microbial properties of the rearing water, natural temporal changes or other environmental factors (Boutin et al., 2013; Sylvain et al., 2016). The rapid community shift observed in the present study highlights that the fish skin-mucus microbiome is susceptible to significant changes within a time frame of 24 h, underling the need of a controlled bacterial community on the skin-mucus of farmed fish (Hoseinifar et al., 2015; Azimirad et al., 2016). Furthermore, we report that the abundance of bacterial biomass obtained from the skin-mucus samples is highly variable. This is an important point to take into account when analyzing skin mucus of water dwelling animals, as samples with low biomass are vulnerable to contamination by the water microbiome.

## Author Contributions

GM carried out the experimental work, collected and analyzed data and wrote the paper. GV-K planned the experiment, analyzed data and contributed to writing the paper. SJ planned the experiment and analyzed data. LH analyzed data and contributed to writing the paper. DP analyzed data (performed statistical analysis). PP analyzed the data and contributed to writing the paper.

## Conflict of Interest Statement

The authors declare that the research was conducted in the absence of any commercial or financial relationships that could be construed as a potential conflict of interest.
