# Supplementary material for: The Skin-Mucus Microbial Community of Farmed Atlantic Salmon (Salmo salar)
Source: Front Microbiol. 2017 Oct 20;8:2043. doi: 10.3389/fmicb.2017.02043 (PMC5655796; doi:10.3389/fmicb.2017.02043)
Supplement: Supplementary file 1 [file Supplementary_Materials.pdf]

Supplementary Material

The skin-mucus microbial community of farmed Atlantic salmon (*Salmo salar*)

Giusi Minniti[1], Live Heldal Hagen[1], Davide Porcellato[1], Sven Martin Jørgensen[2], Phillip B. Pope[1*] and Gustav Vaaje-Kolstad[1*]

[1]Faculty of Chemistry, Biotechnology and Food Science, Norwegian University of Life Sciences (NMBU), Ås, Norway

[2]Nofima AS, Norwegian Institute of Food, Fisheries & Aquaculture Research, Ås, Norway.

*Corresponding authors: Philip B. Pope and Gustav Vaaje-Kolstad

Keywords: skin, mucus, teleost, microbiome, stress, aquaculture, *Salmo salar*

Supplementary Materials and Methods

List of commands and parameters used for the 16S analysis:

Merging:
```
>usearch8 -fastq_mergepairs for_R1.fastq -reverse rev_R2.fastq -
fastqout merged.fastq -fastq_minmergelen 300
```

Quality filtering:
```
>usearch8 -fastq_filter merged.fastq -fastq_maxee 1.0 -fastaout
filtered.fasta
```

The fasta files from all samples were concatenated to one file (also called filtered.fasta) using `cat`.

Identification of unique sequences:
```
>usearch8 -derep_fulllength filtered.fasta -sizeout -fastaout
uniques.fasta
```

Removing singletons:
```
>usearch8 -sortbysize uniques.fasta -fastaout seqs_sorted.fasta -
minsize 2
```

Trimming sequence length:
```
>usearch8 -fastx_truncate seqs_sorted.fasta -trunclen 430 -label_suffix
_430 -fastaout seqs_sorted_430.fasta
```

Removing chimera sequences and clustering:
```
>usearch8 -cluster_otus seqs_sorted_430.fasta -otus otus.fasta -
uparseout out_430.up -relabel OTU
```

Assigning taxonomy and making the OTU table:
```
>usearch8 -utax otus.fasta -db refdbF.ubd -strand both -fastaout
otus_tax_430.fasta

>usearch8 -usearch_global filtered.fasta -db otus_tax_430.fasta -strand
plus -id 0.97 -otutabout otu_table.txt
```

The tab-delimited OTU table was subsequently converted to biom table using biom convert:
```
>biom convert -i otu_table.txt -o otu_table.biom --table-type="OTU
table" --process-obs-metadata taxonomy
```

Filtering out OTUs less than 0.005% of the total sequences in QIIME:
```
>filter_otus_from_otu_table.py -i otu_table.biom -o otu_table_min5.biom
--min_count_fraction 0.00005
```

Performing rarefaction on the OTU table based on the smallest library (22070seqs/sample):
```
>single_rarefaction.py -i otu_table_min5.biom -o
otu_table_min5_even.biom -d 22070
```

QIIME core diversity analyses:
```
>core_diversity_analyses.py -o core_analysis -i
otu_table_min5_even.biom -m mapping_file.txt -c
Category1,Category2,Category3 -e 22070 -t rep_set.tre
```

Supplementary Tables and Figures

Supplementary Table S1. DNA concentrations of mucus and water samples, measured with Qubit fluorometer and the Quant-iT dsDNA HS assay kit. DNA concentrations falling below the minimum quantitative range for the Quant-iT HS assay are noted as not detected (n.d.). The presence (+) or absence (-) of PCR products are specified, in addition to the number of sequences/sample after quality trimming. Samples not included in the analysis are noted as not included (n.i.).

Sample ID	DNA conc. (ng/ml)	PCR	Sequences (n)
M1-0h-1	0.14	+	51521
M2-0h-1	0.33	+	54984
M3-0h-1	0.40	+	41772
M4-0h-1	0.36	+	54460
M5-0h-1	0.40	+	74525
M6-0h-1	0.10	+	50170
M7-0h-1	n.d.	+	47123
M8-0h-1	n.d.	+	29579
M9-0h-1	n.d.	+	45413
M10-0h-1	0.16	+	26697
M11-0h-1	n.d.	+	39172
M12-0h-1	n.d.	-	n.i.
M13-0h-1	n.d.	-	n.i.
M14-0h-1	n.d.	-	n.i.
M15-0h-1	n.d.	-	n.i.
M1-3h-2	0.34	+	43979
M2-3h-2	1.10	+	40594
M3-3h-3	0.23	+	39289
M4-3h-3	0.21	+	56028
M5-3h-3	0.46	+	44608
M6-3h-3	0.12	+	24949
M7-3h-3	0.11	+	57458
M8-3h-3	n.d.	-	n.i.
M9-3h-3	n.d.	-	n.i.
M10-3h-3	n.d.	-	n.i.
M11-3h-3	n.d.	-	n.i.
M12-3h-3	n.d.	-	n.i.
M13-3h-3	n.d.	-	n.i.
M14-3h-3	n.d.	-	n.i.
M15-3h-3	n.d.	-	n.i.
M1-24h-2	0.26	+	30325
M2-24h-2	0.12	+	48690
M3-24h-2	0.22	+	30017
M4-24h-2	0.33	+	69534
M5-24h-2	0.23	+	62707
M6-24h-2	0.21	+	77228
M7-24h-3	0.47	+	54839
M8-24h-3	0.16	+	45539
M9-24h-3	0.20	+	42385

M10-24h-3	0.18	+	68933
M11-24h-3	0.21	+	75218
M12-24h-3	n.d.	+	66863
M13-24h-3	0.31	+	46470
M14-24h-3	n.d.	-	n.i.
M15-24h-3	n.d.	-	n.i.
W1-T0-1	1.23	+	27086
W2-T0-1	2.55	+	34693
W3-T0-1	3.37	+	22070
W1-Ctrl-2	2.05	+	29241
W2-Ctrl-2	3.04	+	57359
W3-Ctrl-2	2.39	+	51639
W1-Ctrl-3	1.13	+	98187
W2-Ctrl-3	2.01	+	62745
W3-Ctrl-3	3.19	+	62536
W1-T3-2	2.97	+	83736
W2-T3-2	4.01	+	85584
W3-T3-2	3.89	+	60564
W1-T3-3	2.10	+	48070
W2-T3-3	2.02	+	48462
W3-T3-3	3.15	+	39664
W1-T24-2	1.99	+	67986
W2-T24-2	4.09	+	63784
W3-T24-2	2.09	+	31459
W1-T24-3	4.00	+	68445
W2-T24-3	3.86	+	32160
W3-T24-3	3.14	+	74109

Supplementary Table S2. Droplet Digital PCR concentration values (target DNA copies/µL) of each replicate (R1, R2, R3) obtained from the QuantaSoft software. These values were used to calculate the number of copies/µL in the initial mucus and water samples at different time points (T0, T3, T24 in addition to water control; Ctrl) as explained in Materials and Methods.

Sample ID	R1	R2	R3	Average±StDev			Copies/µL in initial samples
M1-T0-1	19.55	26.35	31.40	25.77	±	5.95	283
M2-T0-1	162.75	161.35	194.60	172.90	±	18.81	1902
M3-T0-1	550.75	533.35	633.60	572.57	±	53.57	6298
M4-T0-1	336.75	715.35	777.60	609.90	±	238.59	6709
M5-T0-1	610.75	536.35	611.60	586.23	±	43.20	6449
M6-T0-1	62.25	57.65	58.70	59.53	±	2.41	655
M7-T0-1	5.65	4.05	3.60	4.43	±	1.08	49
M8-T0-1	4.85	4.95	4.80	4.87	±	0.08	54
M9-T0-1	8.45	10.85	10.40	9.90	±	1.28	109
M10-T0-1	33.55	26.75	29.00	29.77	±	3.46	327
M11-T0-1	9.35	4.55	3.00	5.63	±	3.31	62
M1-T3-2	161.75	148.35	132.60	147.57	±	14.59	1623
M2-T3-2	163.60	122.60	143.60	143.27	±	20.50	1576
M3-T3-3	115.75	97.35	96.60	103.23	±	10.85	1136
M4-T3-3	280.75	326.60	204.60	270.65	±	61.62	2977
M5-T3-3	511.75	457.35	460.60	476.57	±	30.51	5242
M6-T3-3	39.65	32.95	30.60	34.40	±	4.70	378
M7-T3-3	183.75	190.35	164.60	179.57	±	13.38	1975
M1-T24-2	23.15	14.85	14.70	17.57	±	4.84	193
M2-T24-2	17.75	12.35	13.10	14.40	±	2.93	158
M3-T24-2	8.35	6.55	5.80	6.90	±	1.31	76
M4-T24-2	8.45	15.35	12.60	12.13	±	3.47	133
M5-T24-2	9.55	17.45	19.00	15.33	±	5.07	169
M6-T24-2	15.75	11.05	22.60	16.47	±	5.81	181
M7-T24-3	72.65	26.85	33.90	44.47	±	24.66	489
M8-T24-3	40.05	24.35	27.30	30.57	±	8.34	336
M9-T24-3	26.10	23.00	15.40	21.50	±	5.51	237
M10-T24-3	17.05	16.35	22.00	18.47	±	3.08	203
M11-T24-3	277.75	293.75	315.60	295.70	±	19.00	3253
M12-T24-3	25.45	31.45	32.50	29.80	±	3.80	328
M13-T24-3	12.25	17.25	19.10	16.20	±	3.54	178
W1-T0-1	612.75	535.35	724.60	624.23	±	95.15	6867
W2-T0-1	589.75	560.35	790.60	646.90	±	125.31	7116
W3-T0-1	1657.75	1407.35	2084.60	1716.57	±	342.43	18882
W1-Ctrl-2	793.75	860.35	1008.60	887.57	±	109.98	9763
W2-Ctrl-2	958.75	650.35	1166.60	925.23	±	259.75	10178
W3-Ctrl-2	651.75	681.35	878.60	737.23	±	123.32	8110
W1-Ctrl-3	461.75	424.35	576.60	487.57	±	79.34	5363
W2-Ctrl-3	598.75	555.35	626.60	593.57	±	35.91	6529
W3-Ctrl-3	918.75	1003.35	939.60	953.90	±	44.08	10493
W1-T3-2	882.75	707.35	879.60	823.23	±	100.37	9056
W2-T3-2	1471.75	1379.35	1758.60	1536.57	±	197.76	16902

W3-T3-2	1110.75	972.35	1238.60	1107.23 ±	133.16	12180
W1-T3-3	845.75	838.35	1093.60	925.90 ±	145.28	10185
W2-T3-3	720.75	682.35	954.60	785.90 ±	147.35	8645
W3-T3-3	1276.75	1256.35	1333.60	1288.90 ±	40.03	14178
W1-T24-2	760.75	1242.90	924.60	976.08 ±	245.16	10737
W2-T24-2	1715.75	1490.35	1978.60	1728.23 ±	244.36	19011
W3-T24-2	770.75	630.35	824.60	741.90 ±	100.29	8161
W1-T24-3	1443.75	1231.35	1487.60	1387.57 ±	137.05	15263
W2-T24-3	1357.75	1236.35	1524.60	1372.90 ±	144.72	15102
W3-T24-3	929.75	1324.35	1634.60	1296.23 ±	353.27	14259

Supplementary Table S3. Permutation Multivariate Analysis of Variance (weighted UniFrac distance matrix) of mucus and water samples at different time points.

**Factors**	**P-value**
Mucus: T0 vs. T3	0.012
Mucus: T0 vs. T24	0.001
Mucus: T3 vs. T24	0.001
Mucus at T3: Tank_2 vs. Tank_3	0.650
Mucus at T24: Tank_2 vs. Tank_3	0.082
Mucus time (T0, T3, T24)	0.001
Mucus tanks (Tank_1, Tank_2, Tank_3)	0.011
Mucus vs. water	0.001
T0: water vs. mucus	0.001
T3: water vs. mucus	0.001
T24: water vs. mucus	0.001
Water: T0 vs. T3	0.044
Water: T0 vs. T24	0.214
Water: T3 vs. T24	0.096
Water: T0 vs. Ctrl	0.020
Water: T3 vs. Ctrl	0.016
Water: T24 vs. Ctrl	0.165
Water at T3: Tank_2 vs. Tank_3	0.100
Water at T24: Tank_2 vs. Tank_3	0.500
Water time (T0, T3, T24)	0.186
Water tanks (Tank_1, Tank_2, Tank_3)	0.053

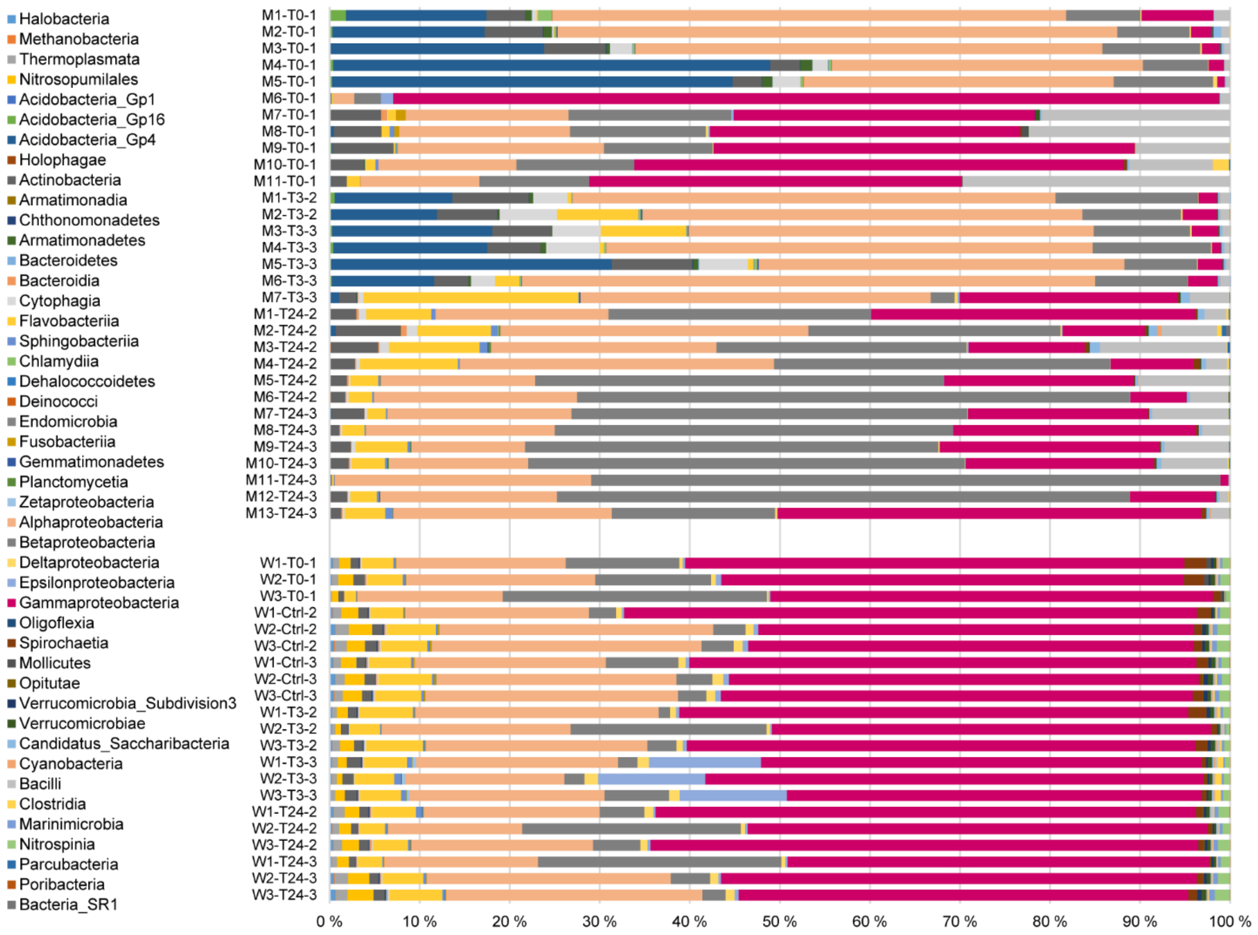

Supplementary Figure S1. Relative abundance of phylotypes at the class level inherent to the mucus and water microbiomes at the different time points (T0, T3, T24 + Ctrl water).
